# Endoscopic Techniques for Lumbar Interbody Fusion: Principles and Context

**DOI:** 10.1155/2022/4979231

**Published:** 2022-03-19

**Authors:** Bryan Zheng, Elias Shaaya, Josh Feler, Owen P. Leary, Matthew J. Hagan, Ankush Bajaj, Jared S. Fridley, Frank Hassel, Raymond Gardocki, Ricardo Casal Grau, Kai-Uwe Lewandrowski, Albert E. Telfeian

**Affiliations:** ^1^Department of Neurosurgery, Warren Alpert Medical School of Brown University, Providence, Rhode Island, USA; ^2^Department of Spine Surgery, Loretto Hospital, Freiburg, Germany; ^3^Vanderbilt University Medical Center, Nashville, Tennessee, USA; ^4^Casal Dots SLU and Asepeyo Hospital, Madrid, Spain; ^5^Center for Advanced Spine Care of Southern Arizona, Tucson, Arizona, USA

## Abstract

Endoscopic techniques in spine surgery are rapidly evolving, with operations becoming progressively safer and less invasive. Lumbar interbody fusion (LIF) procedures comprise many spine procedures that have benefited from endoscopic assistance and minimally invasive approaches. Though considerable variation exists within endoscopic LIF, similar principles and techniques are common to all types. Nonetheless, innovations continually emerge, requiring trainees and experienced surgeons to maintain familiarity with the domain and its possibilities. We present two illustrative cases of endoscopic transforaminal lumbar interbody fusion with a comprehensive literature review of the different approaches to endoscopic LIF procedures.

## 1. Introduction

Endoscopic spine surgery (ESS) techniques for discectomy have been used for decades [1–3], but its first reported use for lumbar fusions was in 1996 [4]. Endoscopic lumbar interbody fusion (ELIF) procedures encompass a group of minimally invasive surgeries (MIS) for treating degenerative disc disease (DDD) of the lumbar spine. Endoscopic spine operations leave a smaller surgical footprint than the minimally invasive surgeries they build upon and further the advantages of MIS, including reduced tissue disruption, decreased intraoperative blood loss, and shorter hospital admissions [5]. Moreover, endoscopic surgeries represent a unique contribution in that they may be performed under conscious sedation instead of general anesthesia [6, 7], engendering potential improvement in patient satisfaction [8, 9]. In the next review article, the authors will discuss the different approaches to LIF and how innovation in endoscopic techniques has driven the learning requirements for these techniques.

## 2. Categories of Endoscopic Lumbar Interbody Fusion

Multiple options for ELIF procedures have emerged within the last half-century. Their respective applications usually parallel those of the traditional lumbar interbody fusion technique from which they were adapted. As such, ELIFs are broadly distinguished by surgical approach, which is the organization employed by this section. A nonexhaustive summary of indications and contraindications for each of the discussed surgery subtypes can be found in Table 1.

### 2.1. Transforaminal Lumbar Interbody Fusion (TLIF)

Initially introduced in 1982 [10], transforaminal lumbar interbody fusion afforded a less invasive alternative to traditional posterior lumbar interbody fusion (PLIF). It was also the first LIF procedure using an endoscopic approach and has since become the most common endoscopic LIF [11, 12]. The first description of an endoscopic TLIF was microendoscopically assisted surgery in a relatively small series of patients [13]. The percutaneous endoscopic TLIF was subsequently reported in an influential publication of 42 cases in 2008 [11]. Both studies confirmed the efficacy and benefits of the endoscopic TLIF, preserving the advantages of MIS, including smaller incisions, less paraspinal muscle dissection, and faster postoperative recovery. The uniportal endoscopic TLIF constitutes the minimum instrument requirements compared to other ELIF procedures. A single working tube is utilized for the endoscope (or microendoscope) and decompression, and the same port is used for implant insertion [14]. Fluoroscopic guidance is typically used to identify anatomy and confirm cage placement as well as to perform pedicle screw fixation, and intraoperative neuromonitoring is commonly used in cases of general anesthesia to avoid injury of nerve roots.

The first and only significant modification to the endoscopic TLIF heretofore came via the unilateral biportal endoscopic TLIF [15]. This technique is very similar to the full-endoscopic uniportal approach, simply separating the working portal from the endoscope, yielding improved ergonomics. Special instruments in comparison to uniportal procedures are not required, as the working and viewing portals are only nominally distinct and can be switched if desired. Additional ports can be added for multilevel cases as well [16]. While a variation of this procedure designated “lordotic endoscopic wedge” ELIF using a unique standalone cage has been published and is purportedly a technically more straightforward approach with comparable short-term outcomes, it is not yet widely performed [17].

### 2.2. Lateral Lumbar Interbody Fusion (LLIF)

There are two lateral approaches for LIF, both of which have been described using an endoscopic approach within the last decade. While there is some ambiguity of terminology, the lateral lumbar interbody fusion (LLIF, sometimes also marketed under the branded acronyms “ELIF” or “XLIF” depending on the equipment used) technique takes a transpsoas approach for indirect decompression of neural elements, which has reduced morbidity over open PLIF and anterior LIF (ALIF) [18, 19]. In a series of 41 patients undergoing LLIF, including six patients undergoing endoscopic-assisted LLIF [20], surgeons reported that endoscopy improved visualization of the genitofemoral nerve overlying the psoas and of the retroperitoneal space, underscoring its potential to minimize complications such as lumbar plexus and visceral injury in LLIF. Though the series was small, the operative and clinical outcomes were equivocal between the two groups. Similarly, in a recent cohort of 70 patients, endoscopic LLIF demonstrated similar efficacy and advantages over open surgery, though no statistical comparison to nonendoscopic XLIF was made [21]. Notably, intraoperative neuromonitoring is particularly important for LLIF procedures including endoscopically guided ones given the potential injury to the lumbar plexus within the psoas major.

The second subset of LLIF is the oblique LIF (OLIF), which takes a prepsoas trajectory and does not present as much risk of injury to the lumbar plexus compared to the transpsoas approach [22]. In a series of 12 patients who underwent endoscopic OLIF, surgeons cited a need to remove disc fragments under direct endoscopic visualization instead of fluoroscopic guidance as to the motivation to use the endoscopy-assisted technique [23, 24]. The endoscope was used principally for the discectomy portion of the procedures. Other hybrid procedures have also been reported. In another study of staged procedures, the combination of biportal endoscopic decompression with OLIF offered comparable clinical outcomes to traditional TLIF [25]. Endoscopic foraminotomy has also been reported as a rescue procedure in patients undergoing conventional OLIF. An adequate margin to the nerve root cannot be identified by EMG, enabling the safe completion of the intended procedure in these patients without dysesthesia due to nerve injury [26].

### 2.3. Anterior Lumbar Interbody Fusion (ALIF)

While considered minimally invasive [27], the anterior lumbar interbody fusion (ALIF) generally does not require an endoscope to aid in the visualization of spinal structures. Still, the ALIF has evolved significantly since it was initially proposed [28], including laparoscopic and miniopen variations [29]. The most relevant endoscopic ALIF procedure is the balloon-assisted retroperitoneal technique for ALIF, a gasless version of the laparoscopic transperitoneal approach [30]. As implied by the designation, special balloon retractors are required in this variation versus common laparoscopic tools and typical carbon dioxide insufflation. While, in theory, the gasless endoscopic approach avoids the risks associated with intentional pneumoperitoneum and has demonstrated efficacy [31], there remains a lack of literature comparing the laparoscopic, endoscopic, and conventional ALIF techniques.

### 2.4. Posterior Lumbar Interbody Fusion (PLIF)

The posterior approach to LIF (PLIF) is usually an open procedure requiring more extensive bony resection than TLIF. Yet, a full-endoscopic PLIF has nonetheless been developed and demonstrated comparable outcomes to minimally invasive TLIF [32]. While the endoscopic PLIF resulted in reduced blood loss, shorter hospitalization, and earlier symptomatic improvement, it is a technically complex procedure that may require some simplification to become widely adopted. As such, the indications for endoscopic PLIF and ALIF are not yet apparent.

## 3. Two Representative Cases of Endoscopic TLIF

### 3.1. Clinical Presentation 1

The patient was a 57-year-old male who had symptoms of left L5 radiculopathy for several years and failed conservative management. His symptoms primarily involved lower back pain on the left that radiated toward the left buttock and hip. However, his physical examination revealed normal strength and sensation throughout the lower extremities.

**Figure 1:**
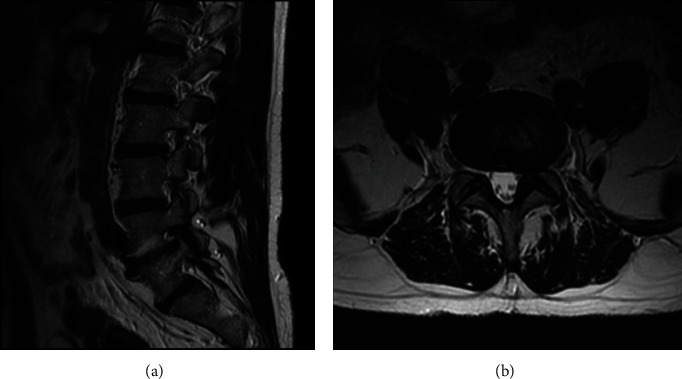
Preoperative T2-weighted MR of the patient who underwent a typical full-endoscopic TLIF. (a) Paramidline sagittal view of lumbar spine from a plane intersecting the left neural foramina, demonstrated moderate stenosis of the L4-5 left neural foramen caused by ligamentum flavum and facet hypertrophy. (b) Axial disc cut at the level of L4-5 further illustrated facet osteoarthritis with compression of the exiting nerve root.

An MRI demonstrated moderate left neuroforaminal stenosis at L4-5 secondary to ligamentum flavum and facet hypertrophy, which also caused mild to moderate narrowing of the central canal (Figure 1). Other findings included further degenerative changes throughout the lumbar spine, including broad-based disc bulges and arthritic changes without significant canal or neuroforaminal stenosis. The patient agreed to undergo full-endoscopic TLIF at L4-5.

### 3.2. Surgical Technique

The patient was intubated then positioned prone on the operating table. General anesthesia was administered to allow for electromyography neuromonitoring, although a monitored anesthesia care (i.e., conscious sedation) protocol may also be utilized and is often preferred in these surgeries.

The skin was marked over the L4-5 level on the left, 10 cm off the midline, such that the angle of entry to the disc space was approximately 45 degrees. An 18-gauge spinal needle was placed in the L4-5 disc space, just entering along the border of the L5 pedicle. A 3 cm incision was made sharply. Sequential dilators were inserted into the incision to dilate the surgical corridor to the disc space. Next, a foraminotomy was performed using a reamer drill system within the ventral portion of the superior articulating.

The final tubular retractor was placed for the decompressive portion of the operation, and a Joimax® endoscope (Joimax GmbH, Germany) was draped and brought into the surgical field. A discectomy was performed, directly visualizing Kambin's triangle (i.e., the exiting nerve root, dural sac, and L5 superior endplate) as well as the traversing nerve roots. These nerve roots were confirmed with neuromonitoring. At this point, a cannula was placed inside the L4-5 disc space and dilated. The discectomy was continued with pituitary, curette, and shaver brushers to denude the endplate. Fluoroscopy was used to confirm the completion of the discectomy.

A RISE® cage (Globus Medical, Audubon, PA) was placed and expanded after the disc space was packed with allograft, confirming positioning under direct endoscopic visualization. Bilateral percutaneous pedicle screws at L4 and L5 were placed via Jamshidi needle and connected with rods, targeting the junction of the transverse process and the facets; then, the caps were securely tightened. Final fluoroscopic radiographs were performed and confirmed the proper position of the screws. The incision was closed in layers. There were no complications associated with any portion of the surgery.

### 3.3. Outcome

**Figure 2:**
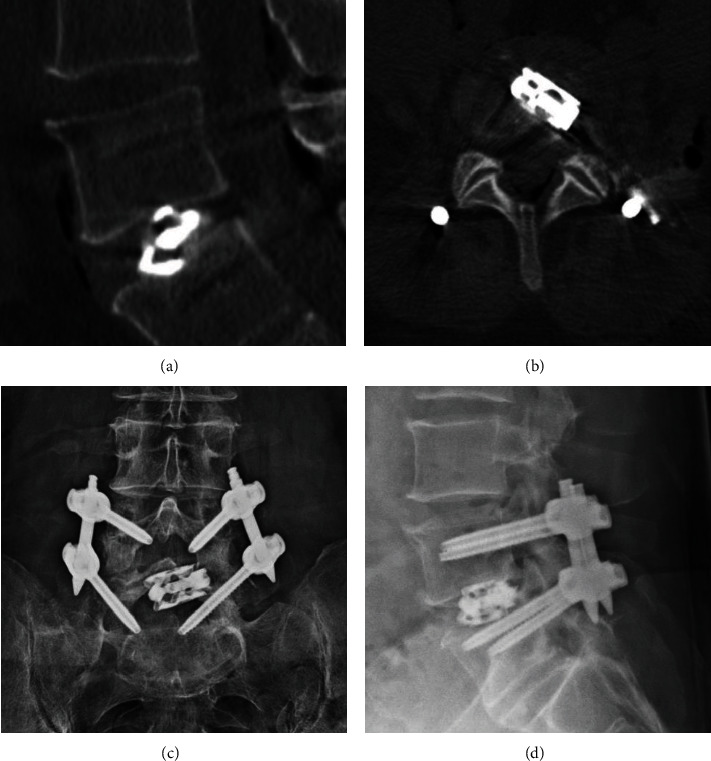
Representative radiographic outcomes of full-endoscopic TLIF. (a) Sagittal CT slice obtained on the day of surgery confirmed adequate graft positioning and L4-5. The expanded cage resulted in disc space recovery, as intended. (b) Associated axial view of implanted hardware revealed expected oblique graft orientation. Procedure-related free air in the retroperitoneum was also noted. (c) Plain anterior-posterior films obtained at short-term follow-up affirmed hardware placement. (d) Associated lateral X-ray also did not reveal evidence of hardware complication.

Postoperative imaging can include plain radiographs or computed tomography (CT) scans of the lumbar spine. In this case, a lumbar spine CT was obtained immediately postoperatively (Figures 2(a) and 2(b)) on the same day, and a spine X-ray was obtained at 6 weeks post-TLIF (Figures 2(c) and 2(d)). Postoperative CT revealed well-positioned hardware, which the plain radiographs confirmed. Neither assessment showed any evidence of hardware complication. At the most recent follow-up, which was six months postoperation, the patient denied any back pain with some residual nerve pain that has been well managed with gabapentin.

### 3.4. Clinical Presentation 2

**Figure 3:**
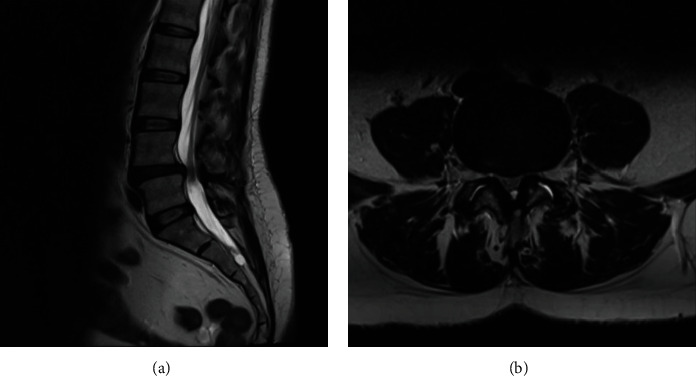
Preoperative lumbar spine T2-weighted MR of a second case illustration. (a) There is grade I anterolisthesis of L4 on L5 and disc herniation resulting in spinal stenosis. (b) Axial slice further demonstrates significant central canal stenosis.

**Figure 4:**
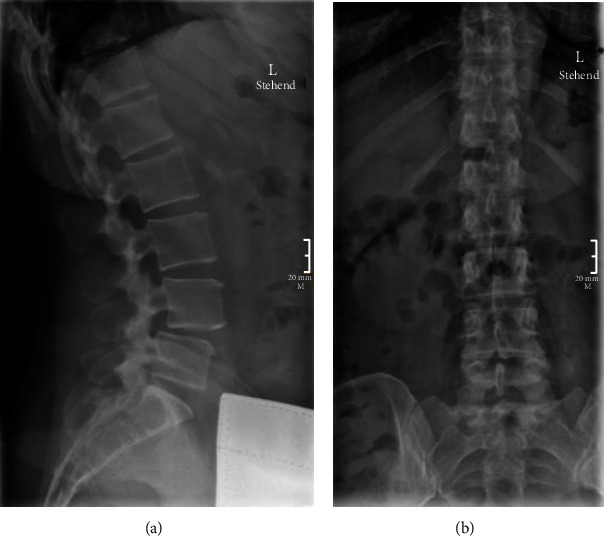
Preoperative X-ray films of lumbar spine in second case illustration. (a) Lateral view highlights spondylolisthesis also seen on MR. (b) AP view is unremarkable.

The patient is a 51-year-old female that presented with years of back pain with right L5 radiculopathy. The patient was full strength with intact sensation in the lower extremities on the exam. They complained of worsening pain with weight-bearing. MRI of the lumbar spine revealed a grade I spondylolisthesis at L4-5 with central canal stenosis (Figure 3). Preoperative X-rays are presented in Figure 4.

### 3.5. Surgical Technique

The patient was brought into the operative suite and placed under general anesthesia. The patient was then positioned prone on the operating table. Electromyographic monitoring was employed throughout the case. X-ray fluoroscopy was used throughout the case for localization.

The skin was marked over the L4-5 level on the right, 10 cm off the midline, such that the angle of entry to the disc space was approximately 45 degrees. An 18-gauge spinal needle was placed in the L4-5 disc space, just entering along the negative border of the L5 pedicle. A 3 cm incision was made sharply. Sequential dilators were inserted into the incision to dilate the disc space. Next, a foraminotomy was performed using a reamer drill system within the ventral portion of the superior articulating. The tubular retractor was placed, and then, a Joimax® endoscope (Joimax GmbH, Germany) was used to visualize Kambin's triangle. Once the L4-5 disc space was identified, a cannula was placed inside and the disc space dilated. The discectomy was continued with pituitary, curette, and shaver brushers to denude the endplate. Fluoroscopy was used to confirm the completion of the discectomy.

Once the discectomy was completed, a nonexpandable Endolif® cage (Joimax GmbH, Germany) was placed and packed with allograft. Direct visualization with the endoscope was performed. Bilateral percutaneous pedicle screws, Percusys® (Joimax GmbH, Germany), at L4 and L5 were placed and connected with a rod. Final fluoroscopic radiographs were performed and confirmed the excellent position of the screws. The incision was closed. There were no complications associated with any portion of the surgery.

### 3.6. Outcome

**Figure 5:**
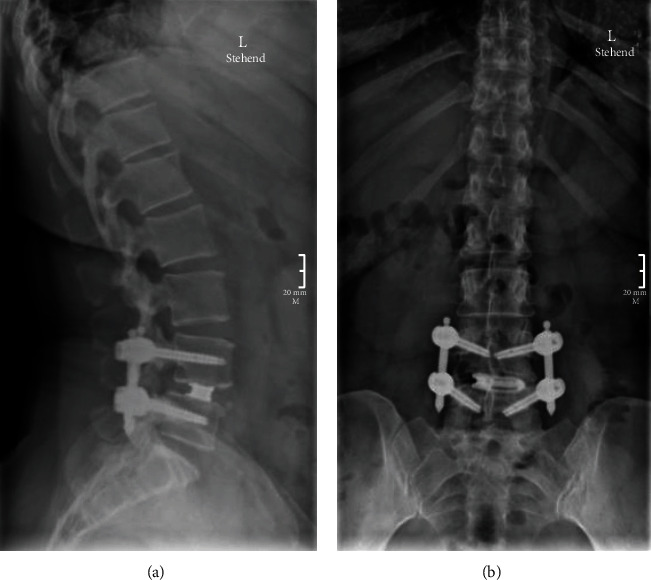
Routine follow-up X-ray status postendoscopic TLIF. (a) Postoperative lateral films demonstrate reduction in L4-5 anterolisthesis with adequate cage placement. (b) Normal percutaneous pedicle screws and interbody cage without evidence of hardware complication.

Postoperative X-rays show the good placement of the interbody cage and pedicle screws with reduction of the spondylolisthesis (Figure 5). The patient is now two years out of surgery. They report that their radicular pain has entirely resolved, and they can walk long distances without difficulty. They remain free of any sensorimotor deficits. Their back pain has improved dramatically.

## 4. Endoscopic Techniques in the Broader Context of Lumbar Interbody Fusions

### 4.1. Clinical Outcomes

Endoscopic TLIF remains the most common and widely used endoscopically assisted LIF. Several prospective studies have characterized the recovery and postoperative course of endoscopic TLIF. Broadly, the recovery period for endoscopic TLIF is shortened relative to the comparable conventional procedure due to decreased approach-related morbidity and pain [31]. In addition, all of the endoscopic LIF procedures discussed previously consistently demonstrate similar complication rates and clinical outcomes to conventional LIF procedures, with both types of surgeries offering excellent relief of symptoms. In a large prospective study comparing endoscopic TLIF with MIS TLIF, there were no differences in outcomes, as measured by the visual analog scale (VAS) for low back and leg pain and Oswestry Disability Index (ODI), 14 months after surgery [33, 34]. A meta-analysis of studies comparing endoscopic and MIS TLIF arrived at the same conclusion. However, it did note faster recovery and earlier postoperative relief of back pain in the endoscopic group [35]. Furthermore, the variations to TLIF, such as the biportal approach, seem to offer equivalent outcomes in the same metrics [36]. In brief, a growing body of evidence has shown comparable clinical outcomes between endoscopic TLIF and MIS TLIF.

### 4.2. Advantages

The endoscopic approach to LIF represents a unique and discrete innovation in spine surgery because they can be performed under local anesthesia with conscious sedation—colloquially termed “awake spinal fusion” [9]. This maneuver avoids the morbidity of general anesthesia, especially in older patients with multiple medical morbidities. It also allows the patient to provide feedback to the surgeon in real time. Recently, this has been of growing interest and has demonstrated favorable outcomes compared to open procedures [8, 37]. Additionally, the awake aspect of endoscopic techniques has been key in the success of endoscopic surgery in the context of a salvage procedure after pseudoarthrosis post-TLIF [38]. However, there are no studies on the differences in long-term outcomes between groups who underwent general versus conscious sedation for endoscopic LIF.

The endoscopic approach reduces the extent of retraction even compared to other minimally invasive procedures, minimizing local tissue injury. The benefits of this approach have been demonstrated with lower levels of serological markers such as creatine kinase and C-reactive protein as well as operative estimated blood loss (EBL) [33]. Moreover, endoscopic techniques further reduce the length of stay associated with MIS surgeries [7, 39, 40]. Though a recent retrospective study failed to corroborate this finding, it did suggest that percutaneous endoscopic LIF was associated with improvements in analgesia and decreased opioid usage, another potential benefit [41]. In a recent review, Wang et al. found that endoscopic TLIF saved the healthcare system an average of $3444 when compared to standard MIS TLIF [40]. The decreased length of stays, decreased morbidity, and decreased analgesic use are all factors in reduced hospital costs.

### 4.3. Limitations

There are limited indications for each kind of TLIF—as such, patient selection is key when considering endoscopic approaches and MIS in general [12]. The primary indications include unilateral foraminal or lateral recess stenosis. However, severe bilateral and/or central canal stenosis, as is seen in many patients with degenerative disc disease, are generally contraindications to percutaneous TLIF. With that said, biportal endoscopic TLIF permits access to the contralateral foramen and may ultimately benefit patients with bilateral stenosis [42].

Secondly, an oft-cited factor when discussing endoscopic spine surgeries is the learning curve involved in adopting such techniques. Indeed, multiple studies have used operative time as a proxy for surgeon comfort. For example, in the context of biportal TLIF, it took 34 cases over approximately 400 days for operative times to plateau [43]. It should be noted, however, that extended learning curves are a theme across MIS spinal procedures and are almost certainly not unique to ELIFs [44–46]. As trainees become familiar with MIS and endoscopic techniques gain wider adoption, surgeon technical expertise and workflow improve with time. Emerging technologies such as augmented reality and other advanced surgical guidance and preoperative planning systems may also help to address such challenges in ESS [47, 48].

## 5. Future Directions

There is a lack of large-scale, multicenter clinical trials comparing endoscopic surgeries with conventional procedures and with each other. The body of literature is mostly limited to case series and medium-sized, single-institutional cohorts. Despite the calls for larger trials, the numerous variations and approaches within endoscopic lumbar fusions cause some disjointedness when attempting to compare outcomes. However, the growing body of literature comparing endoscopic TLIF with MIS TLIF reveals a promising technique with decreased morbidity and cost and comparable patient outcomes.

Endoscopic LIF procedures continue to evolve due to advances in fields such as imaging and navigation. These innovations have helped surgeons overcome unique challenges in complex cases. For example, the combination of intraoperative CT-guided navigation with the transforaminal endoscopic approach was used to decompress heterotopic bone formation after OLIF [49]. More recently, electromagnetic navigation and robot-assisted systems have demonstrated efficient placement of pedicle screws in the context of endoscopic LIF [50]. For instance, a recent case report describes the use of precise imaging fusion technology used to aid puncture in transforaminal endoscopic discectomy [51]. Endoscopic LIFs comprise a group of procedures ripe for the application of innovative technologies.

## 6. Conclusions

Endoscopic LIF procedures are poised to become a mainstay in MIS for lumbar spondylosis as they limit approach-related morbidity, decrease intraoperative blood loss and postoperative pain, limit costs, and improve patient satisfaction. These benefits come at the cost of a surgical view and operating corridor that may be unfamiliar to surgeons without specific training in the technique. As a result, ESS techniques should be included in trainee education with an emphasis on careful patient selection to optimize outcomes. There remains significant work to clarify indications for LIF in general and endoscopic LIF in specific, and standardization of approaches may benefit this goal. As endoscopic approaches to degenerative spinal pathology become more common, improved surgical technique and broadened indications will no doubt emerge, as will greater clarity about the specific risks and benefits of these procedures.

## Figures and Tables

**Table 1:** Possible indications and contraindications for various endoscopic LIF procedures.

		Indications	Contraindications
Endoscopic LIF (any)		(i) Foraminal or lateral recess stenosis (ii) Low-grade spondylolisthesis (I–II)	(i) Bilateral radiculopathy (ii) High-grade spondylolisthesis (III–IV)
TLIF	Percutaneous & microendoscopic	(i) Foraminal or lateral recess stenosis (ii) Low-grade spondylolisthesis (I–II)	(i) Bilateral neuroforaminal stenosis (ii) Severe central canal stenosis
	Biportal TLIF	(i) Bilateral neuroforaminal stenosis	(i) No unique contraindications
LLIF	XLIF	(i) Sagittal, coronal deformity correction	(i) Unfavorable psoas/lumbar plexus/vascular anatomy recognized preoperatively
	OLIF	(i) No unique indications	(i) Intended neuromonitoring
ALIF		Unknown—limited evidence	
PLIF			

## Data Availability

The clinical research data supporting the review sections of this article are from previously reported studies and datasets, which have been cited. The imaging and clinical data used to support the case illustrations are included within the article.
